# Application of multi-extruded fuel injectors for mixing enhancement of hydrogen gas at scramjet engine: computational study

**DOI:** 10.1038/s41598-023-46217-6

**Published:** 2023-11-01

**Authors:** Seyyed Amirreza Abdollahi, Seyyed Faramarz Ranjbar, Saman Aminian, M. Fattahi, P. D. Uyen

**Affiliations:** 1 Faculty of Mechanical Engineering, University of Tabriz, Tabriz, Iran; 2 Department of Civil Engineering, College of Engineering, Cihan University-Erbil, Erbil, Iraq; 3 Institute of Research and Development, Duy Tan University, Da Nang, Vietnam; 4 School of Engineering and Technology, Duy Tan University, Da Nang, Vietnam

**Keywords:** Aerospace engineering, Mechanical engineering

## Abstract

Scramjet engines are considered a highly promising technology for improving high-speed flight. In this study, we investigate the effects of using multi-extruded nozzles on fuel mixing and distribution inside the combustion chamber at supersonic flow. Additionally, we explore the impact of an inner air jet on fuel mixing in annular nozzles. To model fuel penetration in the combustor, we employ a computational technique. Our study compares the roles of three different extruded injectors on fuel diffusion and distribution at supersonic cross-flow. Our findings reveal that the use of an inner air jet increases fuel mixing in the annular jet, while the use of extruded nozzles improves fuel distribution by enhancing the vortices between injectors. These results demonstrate the potential benefits of incorporating multi-extruded nozzles and inner air jets in the design of scramjet engines.

## Introduction

The development of scramjet engines has received considerable attention in recent years due to their potential to improve high-speed flight^1–3^. One of the key challenges in designing these engines is achieving efficient fuel mixing and distribution at supersonic flow within the combustion chamber. In this context, the present study investigates the use of multi extruded nozzles to enhance fuel mixing and distribution. The study also explores the impact of an inner air jet on fuel mixing in annular nozzles. The computational modeling technique is employed to analyze the fuel penetration and diffusion in the combustor^4,5^. The study compares the performance of three different extruded injectors in terms of fuel diffusion and distribution at supersonic cross flow. The findings reveal that the use of extruded nozzles and inner air jet can significantly improve fuel mixing and distribution by enhancing the vortices between injectors. The results of this study have important implications for the design and development of more efficient and effective scramjet engines^6–8^.

Scramjet engines have been a topic of extensive research due to their potential to achieve high-speed flight and space access^9–11^. The critical challenge in designing these engines is to achieve efficient fuel mixing and distribution, as the combustion process is highly sensitive to these factors^12–14^. One way to improve fuel mixing and distribution is by using multi-nozzle injectors. A study^15^ investigated the use of multi-jet injectors in a Mach 6 scramjet combustor and found that the use of multi-jet injectors improved the combustion efficiency and reduced the flame length.

Another approach to improve fuel mixing and distribution is by using transverse injection, where fuel is injected perpendicular to the air flow. Several studies^4,16,17^ investigated the use of transverse injection in a Mach 4 scramjet engine and found that it significantly improved fuel mixing and distribution.

The use of swirling flow to enhance fuel mixing and distribution has also been explored. Many papers^18–21^ investigated the use of swirling flow in a Mach 2.5 scramjet engine and found that it improved fuel mixing and distribution by promoting turbulence and increasing residence time. The impact of fuel injection pressure on fuel mixing and distribution has also been studied. Many papers^22–26^ investigated the effect of fuel injection pressure on the combustion efficiency of a Mach 3 scramjet engine and found that higher injection pressures improved fuel mixing and distribution, resulting in higher combustion efficiency.

The impact of nozzle shape and geometry on fuel mixing and distribution has also been explored. Many papers^26–29^ investigated the effect of nozzle geometry on the combustion efficiency of a Mach 5 scramjet engine and found that the use of lobed injectors improved fuel mixing and distribution compared to circular injectors.

In summary, there have been various approaches to improving fuel mixing and distribution in scramjet engines, including the use of multi-nozzle injectors, transverse injection, swirling flow, higher injection pressures, and optimized nozzle geometry^30–33^. These studies provide valuable insights for the design and development of more efficient and effective scramjet engines^34–36^.

**Figure 1:**
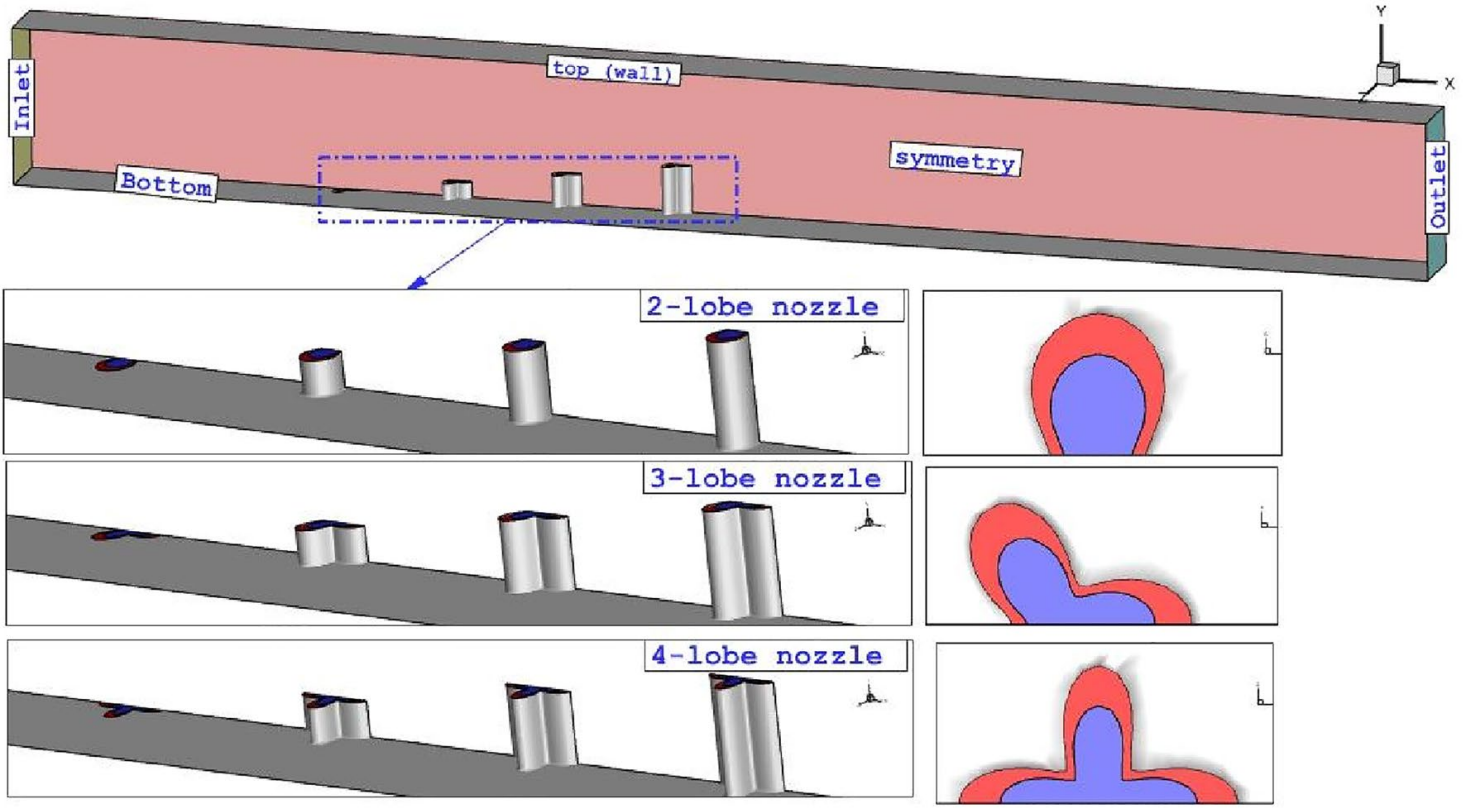
Problem description.

This study has tried to investigate the influence of different arrangements of extruded lobe-injectors on the fuel penetration and shock interactions inside the combustion chamber. To do this, shock waves are compared on the jet planes to reveals the influence of extruded configurations on the fuel mixing mechanism at supersonic flow. Various lobe nozzles are investigated as demonstrated in Fig. 1. The effects of both annular and coaxial fuel and air jet are fully investigated in this research.

## Governing equations and numerical methodology

The simulation of high-speed air stream with compressibility effects is mainly done via solving RANS equations for continuum domain^37–40^. The shock wave formation is inherently happened in our model and consequently, the energy equation must be considered in our modeling^41,42^. Meanwhile, the turbulence effects are also important in our model and SST model of turbulence is considered for the calculation of viscosity^31,42,43^. The secondary gas of hydrogen is also modeled as fuel jet and mass transport equation is also used with Fick. Law for estimation of the diffusion of hydrogen gas^44,45^. Reactions are not considered in this study. The air flow is considered as ideal gas in the present model^46–51^.

Figure 1 illustrates the shape of the injector and arrangement of the nozzle inside the model. The fuel jet is injected through annular nozzle while air jet is released from inner core of the nozzle. The size of outer and inner nozzle is equal and equivalent to circular nozzle with diameter of 0.5 mm. The jet distance of these injector in all configurations is 3.5 mm and the height of extruded nozzle of 2nd, 3rd and 4th nozzle is 0.5 mm, 1 mm and 1.5 mm. The length, width and height of computational domain are 80 mm, 5 mm and 6 mm, respectively. At inlet, air stream with Mach number of 4 and static temperature of 1000 K is applied. The fuel and air jets are released with Mach = 1 and 10% total pressure of supersonic air stream. Half of real model is simulated to reduce the computational cost^52^. The usage of theoretical method is conventional in engineering applications^53–56^.

**Figure 2:**
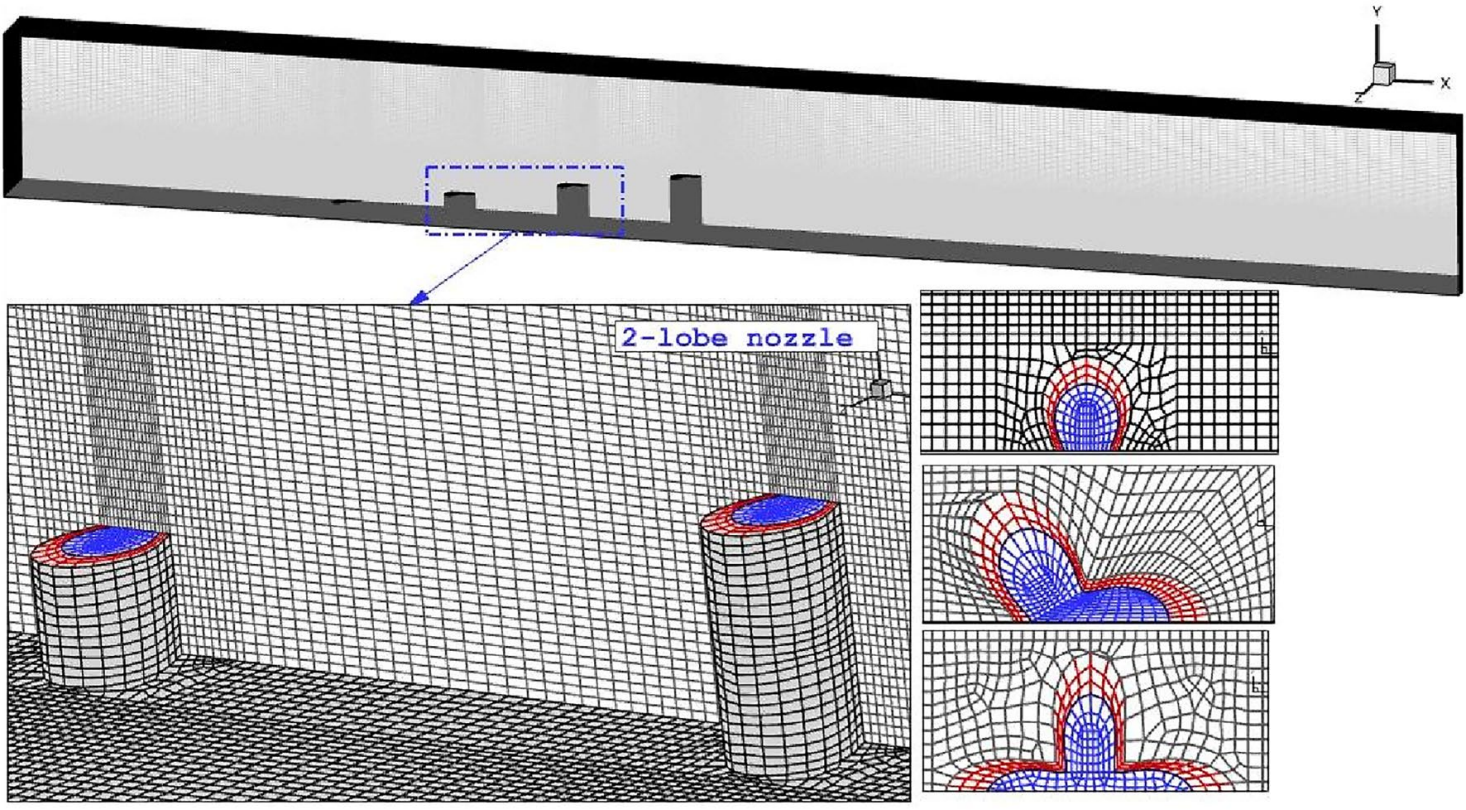
Grid production.

The produced grid for the selected computational domain is displayed in Fig. 2. The structured grid is applied for the model because of the shock interactions. Besides, the resolution of generated grid must be high enough in the regions nearby the nozzle since these districts are potential for the high velocity and density gradient. The grid study is also done to obtain the proper size of grid for the present investigations. Table 1 presents the results of grid independency analysis and it is noticed that the fine grid is most efficient grid for the chosen model.

**Table 1:** Grid study.

	Cells	Grid cells. along X, Y and Z direction	Hydrogen fraction at 30 mm downstream
Coarse	943,100	176 × 114 × 50	0.292
Medium	1,610,100	196 × 144 × 60	0.301
Fine	2,223,100	218 × 170 × 70	0.307
Very fine	3,800,100	242 × 200 × 80	0.308

## Results and discussion

**Figure 3:**
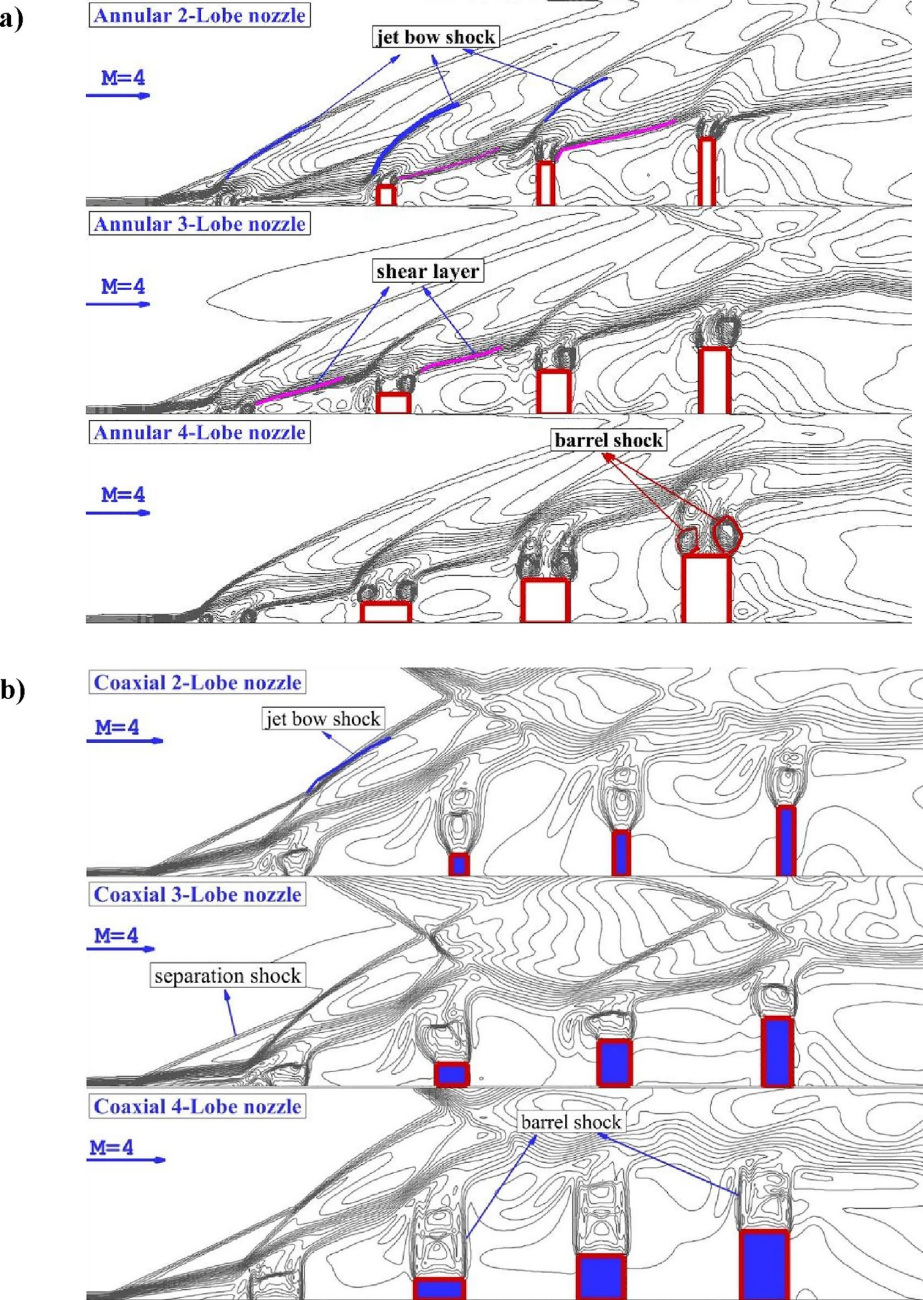
Comparison of Mach contour for (**a**) annular jet system (**b**) coaxial jet.

The correctness of the achieved data from the computational study is evaluated to certify the precision of the applied approach. Figure 3 displayed the value of fuel penetration of single circular nozzle with diameter of 2 mm at supersonic flow. These outcomes advocate that the single-jet numerical solution is accurate in terms of the penetration downstream of jet^38^ (Table 2).

**Table 2:** penetration height (mm).

Distance from injector (mm)	Our data	Numerical data of Fallah^11^	Errors (%)
5	5.68	6.3	10
10	7.05	7.5	6
20	8.05	7.7	4.5
30	8.35	8.5	2.5

Figure 3a illustrates the primary feature of shock waves layer near these annular injectors at supersonic flow on the symmetry plane. The Mach contour of the proposed configuration reveals that the shear layer and jet bow shack significantly varies by the shape of injector on the jet plane. Indeed, the jet bow shock, which is produced by the velocity of fuel jet, deformed by the incoming air stream. When the simple injectors are used, the growth of shear layer is related to the total pressure of jet and free stream condition. However, in extruded jet configuration, the jet plume is related to the height of extruded nozzles. The difference of the shock layer for the model with inner air jet (Fig. 3b) with former annular cases (Fig. 3a) indicates that the high pressure of the coaxial fuel and air jet released from the extruded nozzle results separation shock upstream of the first nozzle. Hence, the formation of the barrel fuel jet is not changed under influence of the incoming supersonic flow.

**Figure 4:**
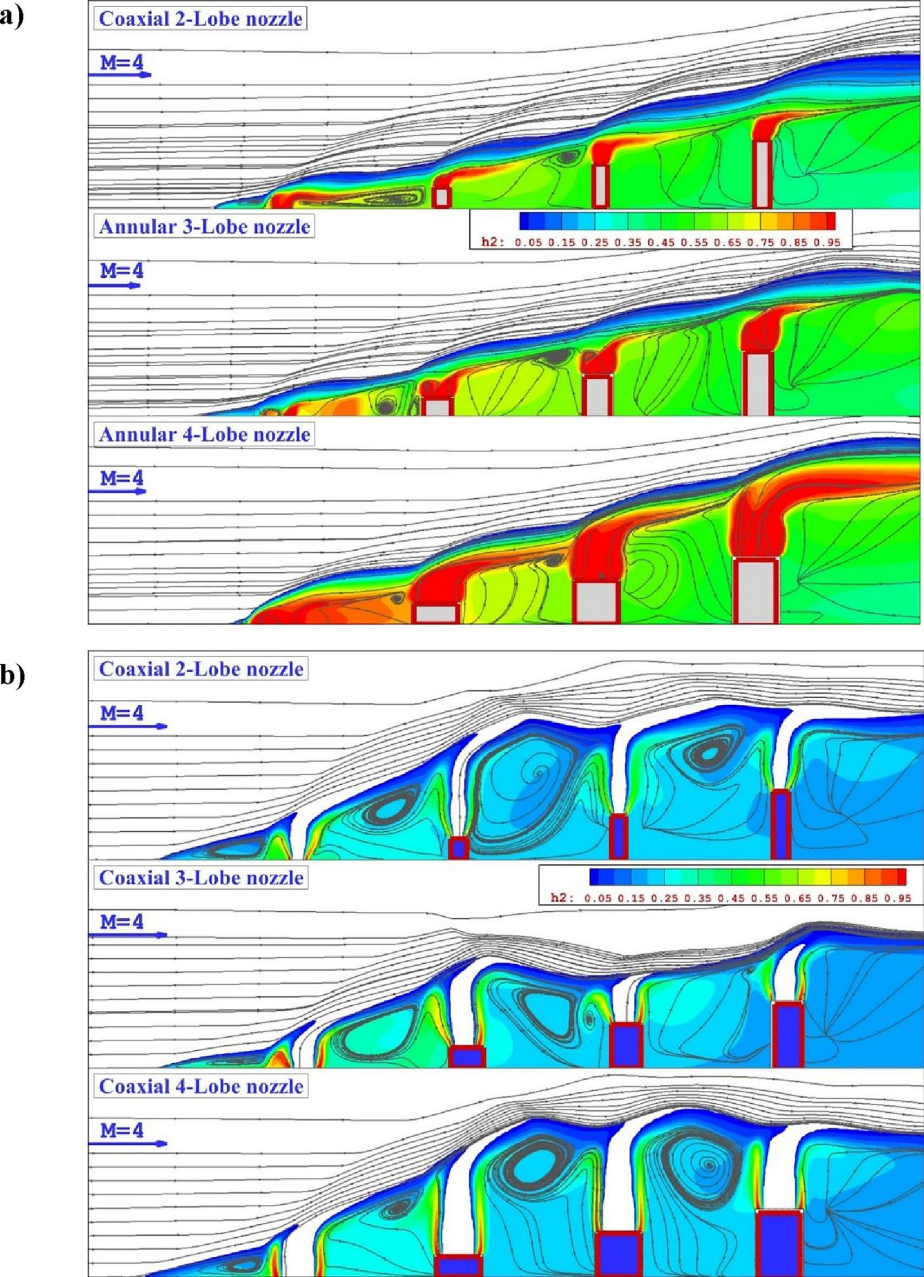
Comparison of flow stream and fuel concentration on the symmetry plane for (**a**) annular jet system (**b**) coaxial jet.

Figure 4a illustrates the contour of mass on the symmetry plane for different annular lobe-nozzles. When the extruded injector is applied for the fuel mixing, the height of the fuel mixing zone is managed by the height of the extruded injectors. As confirmed by the mass contour, the concentration of the fuel jet and penetration height is directly varied by the nozzle height. Besides, the interactions of the fuel jet with main stream almost constant for all jets. In fact, the main advantageous of this concept is management of the fuel diffusion and the efficient fuel diffusion by the jet interactions even in last injectors. As depicted in Fig. 4b, the addition of inner air jet also has meaningful impacts on the concentration and diffusion of the fuel jet in proposed configurations. Comparison of coaxial nozzle with annular one shows that the usage of inner air flow substantially improves the circulation in the gap of injector which enhance the fuel mixing and diffusion of the fuel in the depth of the domain. Indeed, the fuel mixing is more efficient in this configuration.

**Figure 5:**
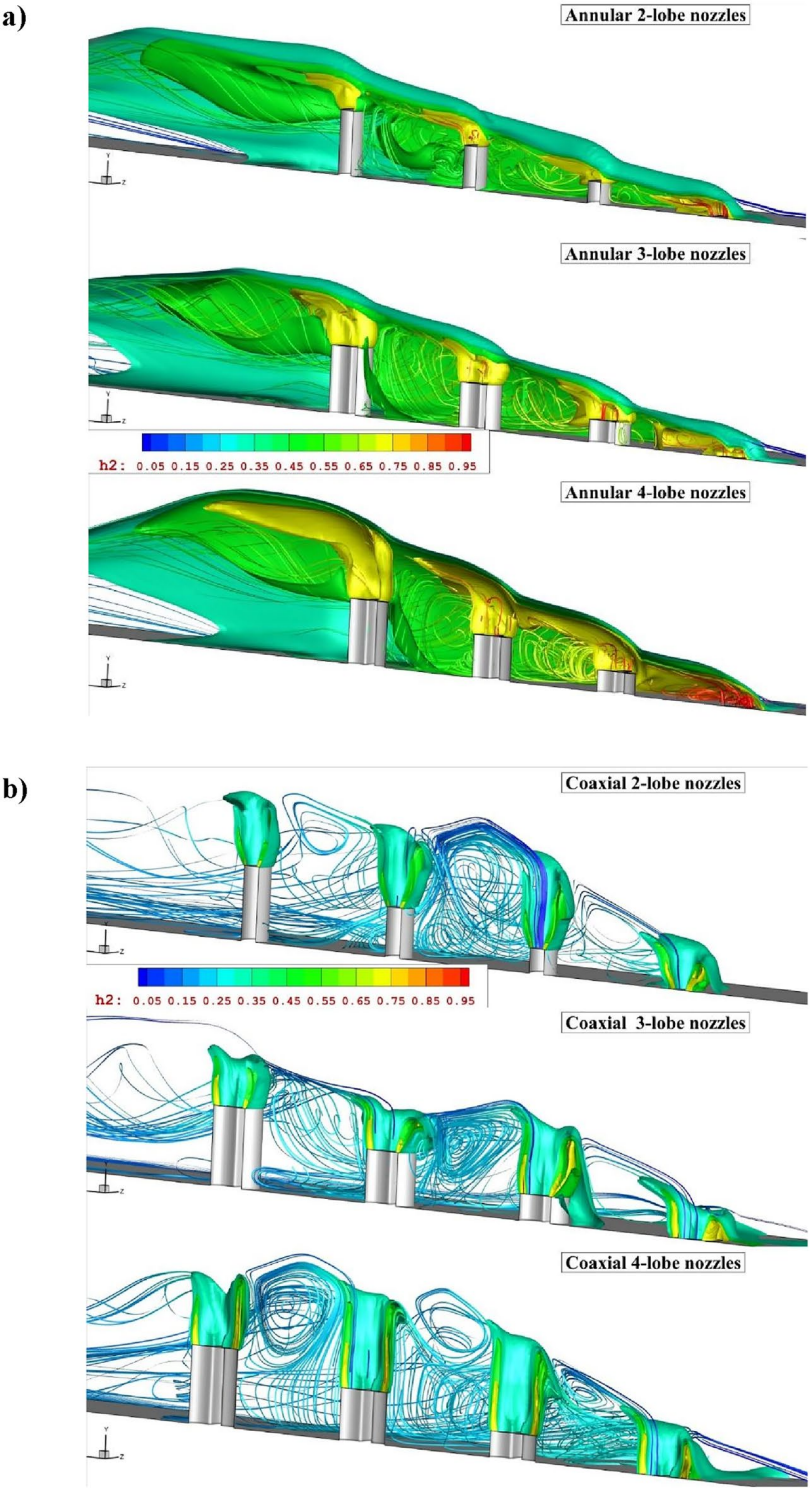
Comparison of three-dimensional flow near the extruded nozzle for (**a**) annular jet system (**b**) coaxial jets.

The three-dimensional flow feature of annular jet configurations (Fig. 5a) disclosed the influence of the circulation in the gap of the injectors on the jet layer at supersonic flow. The comparison of different lobe-nozzle types shows that the fuel distribution inside the main stream is improved by the nozzle with 4-lobe shape. In facts, the fuel jet released from wider area in 4-lobe nozzles and this extend the fuel plume inside the combustion chamber. The injection of the air jet in the recommended jet arrangements (Fig. 5b) shows that the jet interactions are extremely amplified when inner air jet is coupled with annular fuel jets. Meanwhile, the effects of the upstream circulation on the distribution of the fuel jet is more pronounced in the coaxial air and fuel injection.

**Figure 6:**
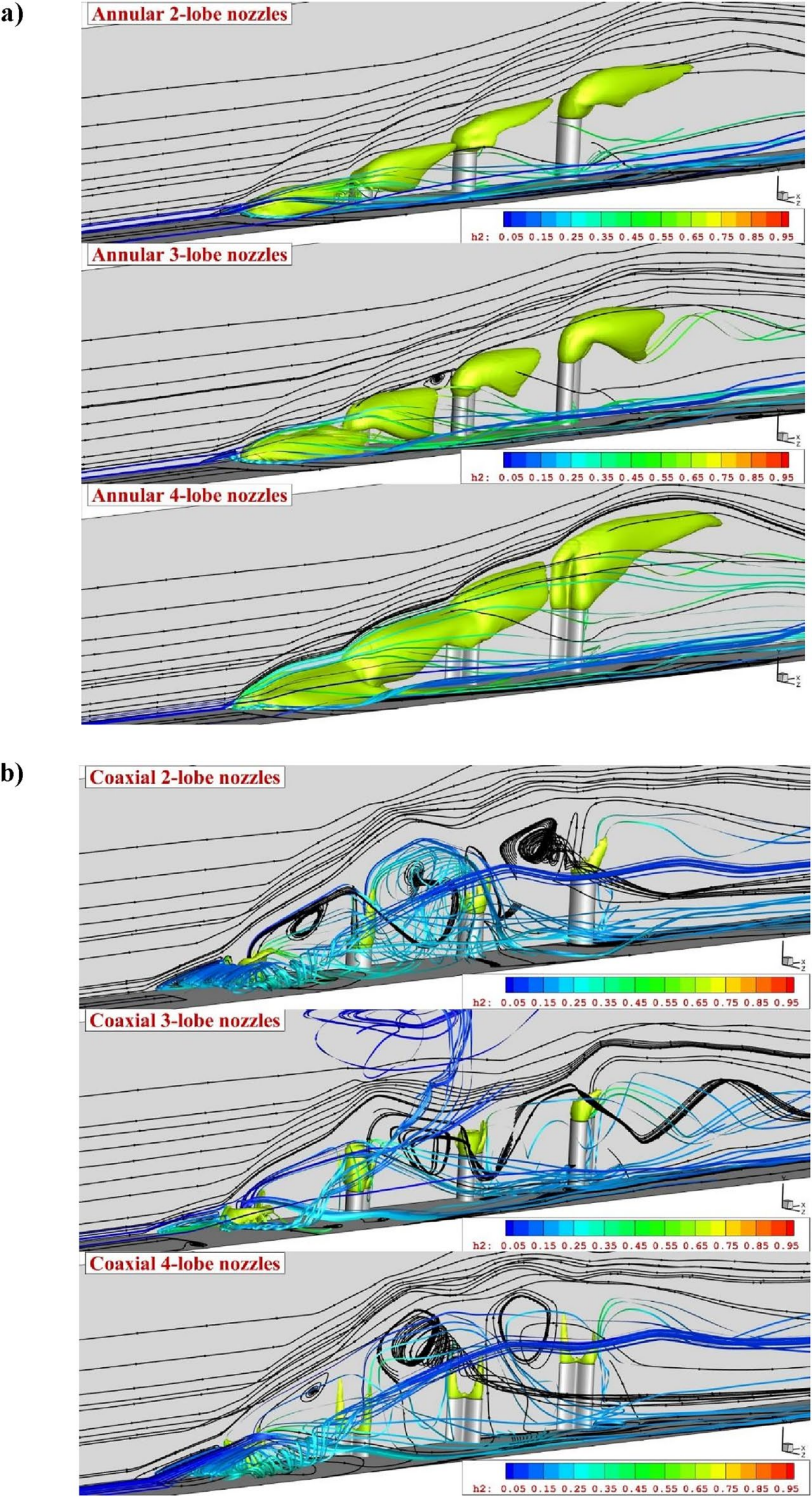
Comparison of flow and jet interactions for (**a**) annular jets (**b**) coaxial jets system.

In Fig. 6, the jet stream along with fuel jet is investigated and displayed to reveal the importance of jet interactions on the mechanism of fuel diffusion in combustion chamber for the proposed models. As mentioned in the previous sections, the interactions of the air and fuel jet becomes extraordinary when extruded multi-nozzles is used in supersonic flow. As demonstrated in Fig. 6a, each jet has its own interactions with air stream in the extruded configurations and the formation of the vortex within the gap of extruded nozzle is extended by the height of the fuel nozzle. These two features are considerably important for the distribution of the fuel multi-jets. As illustrated for the coaxial jet configurations (Fig. 6b), higher jet interactions are achieved by adding inner air jet and this extend the upstream circulations. Therefore, horseshoe vortex with stronger circulation extend to downstream. As the normal momentum of the fuel and air jets increases in this model, the pressure in the gap of nozzle is extended and this enables the formation of the vortex in this region. Therefore, the fuel distribution is more uniform in this configuration.

**Figure 7:**
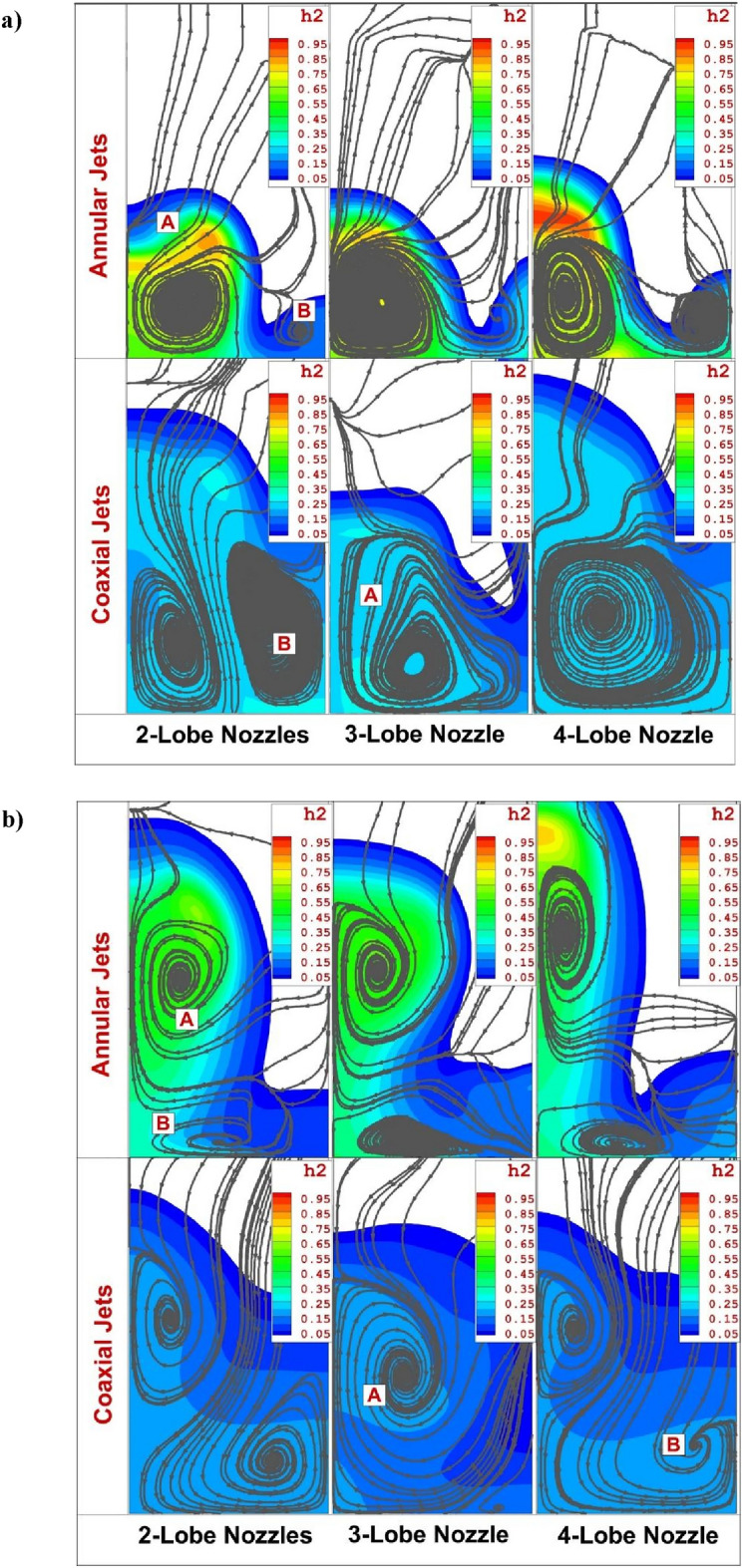
Comparison of mass concentration and stream of annular and coaxial jets at (**a**) X/D = 10, (**b**) X/D = 30 downstream of first jet.

Comparison of the mixing zone and stream feature on two planes (x/D = 10 and 30) down stream of three suggested nozzle configurations are done in Fig. 7. In the vicinity of nozzle (Fig. 7a), the height of fuel penetration is less for annular injector while higher fuel penetration is noticed for the coaxial model. There are two main vortices behind the jet and strength and size of these vortices clearly disclose the role of injector type and existence of inner air jet in the planned configurations. The main circulations (Vortex A) is inherently produced by the normal velocity of fuel jet and second one (Vortex B) is related to the horseshoe vortex. In some nozzle the secondary vortex is not observed especially in the coaxial model. In the far distance (Fig. 7b), the position of these two vortices are changed and the concentration of the fuel gas becomes uniform by the usage of inner air jet.

**Figure 8:**
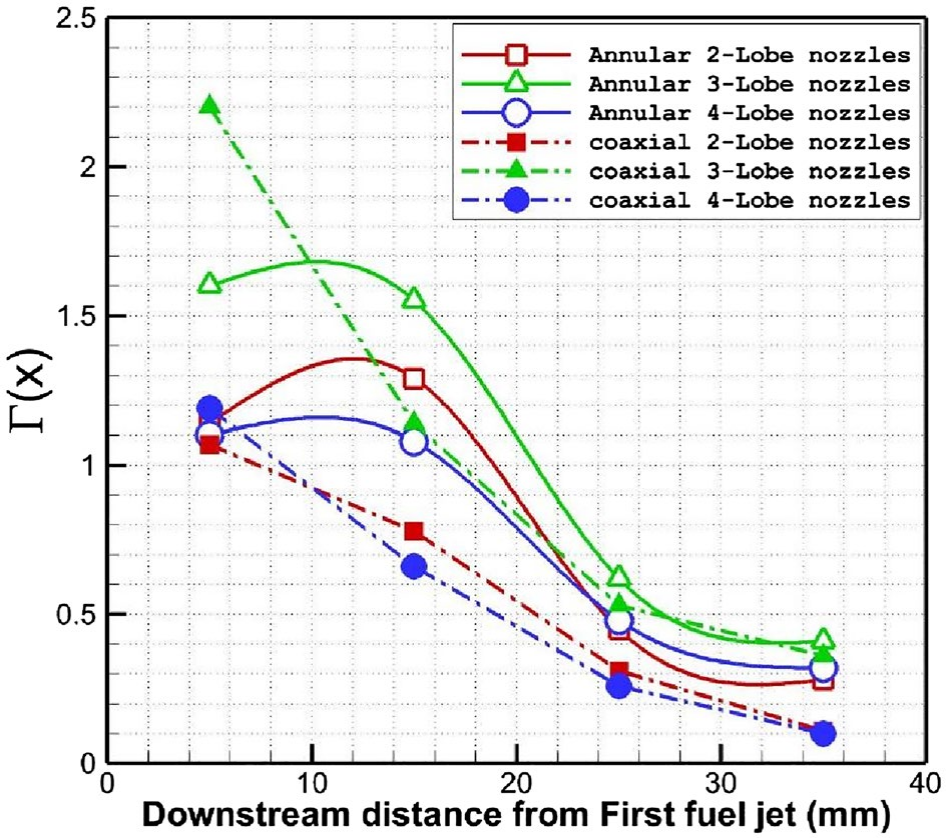
Comparison of the circulation induced by jet type downstream of the injectors.

Comparison of the circulation strength downstream of the proposed configurations is presented in Fig. 8. The variation of the circulation power indicates that replacing the annular jet with coaxial air and fuel jet decreases the circulation power in all models. Among these injector types, 3-lobe model has maximum circulation power and this is due to the formation of larger upstream circulation. The data of circulation shows that the impacts of injector types and nozzle is almost in range of 10 mm to 20 mm behind the first nozzle.

**Figure 9:**
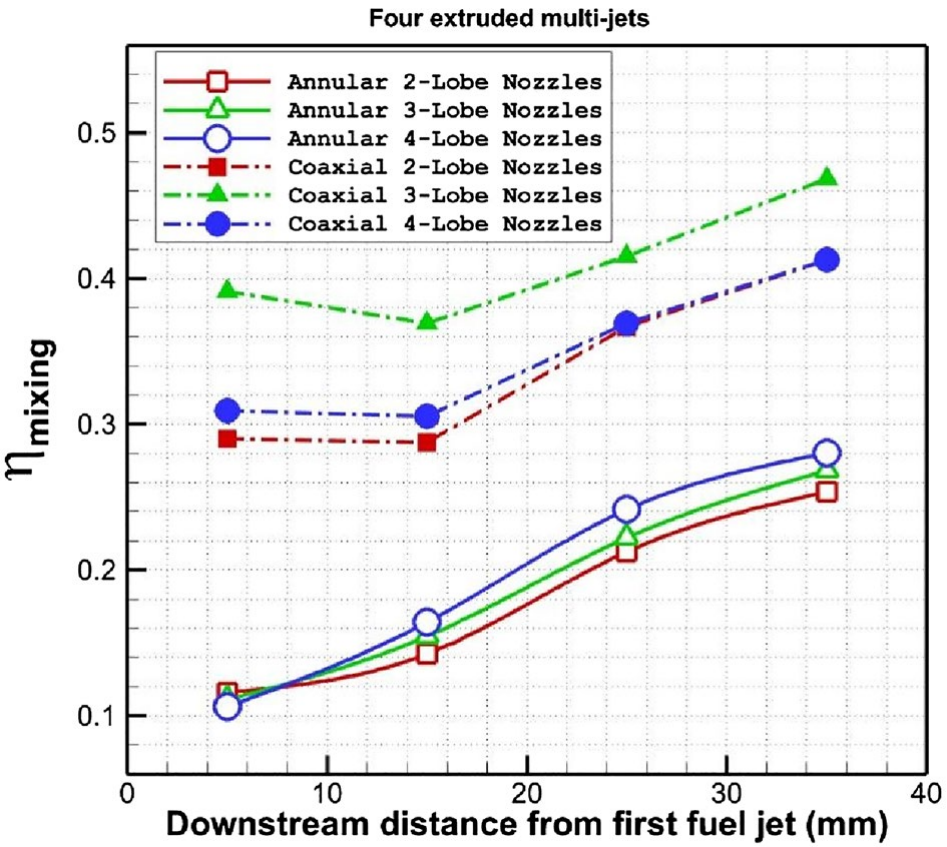
Comparison of the fuel mixing efficiency downstream of the injectors.

Figure 9 plots the changes of fuel mixing behind the fuel nozzles for the proposed extruded injectors. The mixing efficiency of coaxial air and fuel jet is almost 44% more than annular nozzle. The main performance of injector type is noticed near the injectors. In the coaxial nozzle configurations, the 3-lobe nozzle performs more efficient in fuel distribution than other injector configurations.

## Conclusion

This study focuses on investigating the effects of multi extruded injectors on the fuel mixing mechanism in scramjet engines. Specifically, we examine the roles of three different types of nozzles located inside the engine's combustor, including annular and coaxial nozzles. To model the release of fuel jet from the extruded nozzles at supersonic cross flow, we develop a computational fluid dynamic approach. Through this approach, we compare the mixing efficiency and circulation power of each model. Our findings indicate that the use of coaxial jet nozzles decreases the circulation power, while achieving maximum fuel mixing efficiency. Additionally, we observe that the utilization of an inner air jet in the coaxial configuration further enhances fuel mixing in the nozzle gap.

## Data Availability

All data generated or analysed during this study are included in this published article.
